# Association between statin use and the risk of gout in patients with hyperlipidemia: A population-based cohort study

**DOI:** 10.3389/fphar.2023.1096999

**Published:** 2023-02-15

**Authors:** Guan-Ling Lin, Hsiu-Chen Lin, Hsiu-Li Lin, Joseph Jordan Keller, Li-Hsuan Wang

**Affiliations:** ^1^ School of Pharmacy, College of Pharmacy, Taipei Medical University, Taipei, Taiwan; ^2^ Department of Pediatrics, School of Medicine, Taipei Medical University, Taipei, Taiwan; ^3^ Department of Clinical Pathology, Taipei Medical University Hospital, Taipei, Taiwan; ^4^ Department of Neurology, General Cathay Hospital, Sijhih Branch, New Taipei City, Taiwan; ^5^ Department of Psychiatry, Western Michigan University Homer Stryker M.D. School of Medicine, Kalamazoo, MI, United States; ^6^ Department of Pharmacy, Taipei Medical University Hospital, Taipei, Taiwan

**Keywords:** statins, gout, hyperlipidemia, anti-inflammatory, dose-dependent, duration-dependent

## Abstract

**Objective:** To investigate the association between statin use and risk of gout in patients with hyperlipidemia.

**Methods:** In this population-based retrospective cohort study, patients ≥20 years and diagnosed as having incident hyperlipidemia between 2001 and 2012 were identified from the 2000 Longitudinal Generation Tracking Database in Taiwan. Regular statin users (incident statin use, having 2 times and ≥90 days of prescription for the first year) and two active comparators [irregular statin use and other lipid-lowering agent (OLLA) use] were compared; the patients were followed up until the end of 2017. Propensity score matching was applied to balance potential confounders. Time-to-event outcomes of gout and dose- and duration-related associations were estimated using marginal Cox proportional hazard models.

**Results:** Regular statin use non-significantly reduced gout risk compared with irregular statin use (aHR, 0.95; 95% CI, 0.90–1.01) and OLLA use (aHR, 0.94; 95% CI, 0.84–1.04). However, a protective effect was noted for a cumulative defined daily dose (cDDD) of >720 (aHR, 0.57; 95% CI, 0.47–0.69 compared with irregular statin use and aHR, 0.48; 95% CI, 0.34–0.67 compared with OLLA use) or a therapy duration of >3 years (aHR, 0.76; 95% CI, 0.64–0.90 compared with irregular statin use and aHR, 0.50; 95% CI, 0.37–0.68 compared with OLLA use). Dose- and duration-dependent associations were consistent in the 5-year sensitivity analyses.

**Conclusion:** Although statin use was not associated with a reduction in gout risk, the protective benefit was observed in those receiving higher cumulative doses or with a longer therapy duration.

## 1 Introduction

Gout is the most common cause of inflammatory arthritis. Hyperuricemia is the first phase in the pathogenesis of gout. When the urate level exceeds the saturation point, crystallized monosodium urate (MSU) is formed and deposited in the articular or periarticular tissue (Schett et al., 2015; Dalbeth et al., 2021). This pathological crystal can act as a danger signal, trigger the innate immune response, and subsequently cause an inflammatory gout flare (Gong et al., 2020). In 2020, a global epidemiology survey indicated that the prevalence of gout ranged from <1% to 6.8% and the incidence of gout was 0.58–2.89 per 1,000 person-years. The prevalence of gout is higher in men and increases with age (Dehlin et al., 2020). An earlier survey showed that the prevalence and incidence of gout tended to be higher in developed countries than in developing countries. The highest prevalence was observed in Pacific countries and some ethnic groups; the prevalence of gout exceeded 10% in Taiwanese aboriginals and Maori (Kuo et al., 2015b). A Taiwanese nationwide survey indicated that the prevalence and incidence of gout were 6.24% and 2.74 per 1,000 person-years, respectively. Although both decreased from 2005 to 2010, they are still higher compared with those worldwide (Kuo et al., 2015a).

Soluble urate, identical to crystallized MSU, can trigger inflammation, but involves different types of immune cells and molecular mechanisms (Cabău et al., 2020). Both MSU and soluble urate are associated with a higher incidence of cardiovascular diseases, chronic kidney disease, metabolic syndrome, diabetes mellitus (DM), aging, and cancer (Yu et al., 2018). Gout can increase the risk of mortality in patients with cardiovascular diseases and coronary heart disease (CHD) (Clarson et al., 2015). Moreover, gout is prone to cluster together with renal diseases, metabolic syndrome, and cardiovascular diseases; however, the causal relationship or the bidirectional association remains to be clarified (Sumpter et al., 2020).

Statins are the most commonly used, safe and inexpensive antihyperlipidemic agent. Apart from cholesterol reduction, statins exhibit anti-inflammatory pleiotropy. By inhibiting HMG-CoA reductase, statins reduce the biosynthesis of intermediate isoprenoids, such as farnesyl pyrophosphate and geranylgeranyl pyrophosphate, and thus diminish downstream small GTP-binding proteins (e.g., Rho and, Rac), which are associated with the transcription of nuclear factor (NF)-κB-related inflammatory components (Jain and Ridker, 2005). Statins can reduce the morbidity or mortality of patients with certain immune (Khattri and Zandman-Goddard, 2013; Zeiser, 2018; Dehnavi et al., 2020) and inflammatory (Côté-Daigneault et al., 2016; So et al., 2018; Lei et al., 2020) diseases and thus might be repurposed for clinical use. Both MSU and cholesterol can form pathological crystals, and share a common pathway, called the two-signal pathway, to trigger inflammatory responses and cytokine release (So and Martinon, 2017; Koushki et al., 2020). Statins inhibit not only ligand-receptor binding, signal transduction, and inflammatory cytokine production in the 2-signal pathway (Bahrami et al., 2018), but cholesterol crystal-induced inflammation in atherosclerosis (Parsamanesh et al., 2019; Koushki et al., 2020). We hypothesize that statins would inhibit the MSU-induced gout flare through its anti-inflammatory property. This nationwide population-based cohort study determined whether statins have chemopreventive potential against gout.

## 2 Methods

### 2.1 Data sources

Taiwan’s National Health Insurance (NHI) program covered more than 99.9% of Taiwan’s population until 2018 (Lin et al., 2018). The claims data of NHI beneficiaries are collected and added to databases managed by the Health and Welfare Data Science Center (HWDC) of the Ministry of Health and Welfare. This study used the 2000 Longitudinal Generation Tracking Database (LGTD 2000), a randomly sampled dataset comprising the data of 2 million NHI beneficiaries. The LGTD 2000 contains deidentified data regarding insured persons’ demographic variables (e.g., sex and age), outpatient visit or inpatient care, disease diagnoses, and prescriptions details and is considered to be nationally representative for the 23 million residents of Taiwan (Lin et al., 2018). The Registry for Beneficiaries and Cause of Death Data were used to accurately calculate age and obtain the death dates of patients. To protect patients’ privacy, all databases released by the HWDC are anonymous and encrypted. Any individual’s identity cannot be identified. Our study was approved by the Joint Institutional Review Board of Taipei Medical University, Taipei, Taiwan (TMU-JIRB No. 202107064). A waiver of the requirement for patients’ informed consent was granted for this study.

### 2.2 Study design and patients

This study adopted a population-based retrospective cohort design. We enrolled the incident cases of hyperlipidemia [*International Classification of Diseases (ICD), Ninth Revision, Clinical Modification (CM*), *ICD-9-CM,* code: 272.X] recorded at least three times in outpatient clinics or once during hospitalization between 1 January 2001, and 31 December 2012. The index date was set as the first prescription date of statin use in both regular and irregular statin users or other lipid-lowering agent (OLLA) use in OLLA users. To ensure an incident case, we used a washout period of at least 1 year before the diagnosis date. To define new users and examine baseline characteristics, we used a washout period of at least 1 year before the index date. The follow-up was initiated from the index date until gout diagnosis, death, or the end of the study (31 December 2017), whichever occurred first. To investigate the effect of attrition bias, a sensitivity analysis was performed. We considered a follow-up period of 5 years for each individual instead of the period from the index date to the end of the study.

**FIGURE 1:**
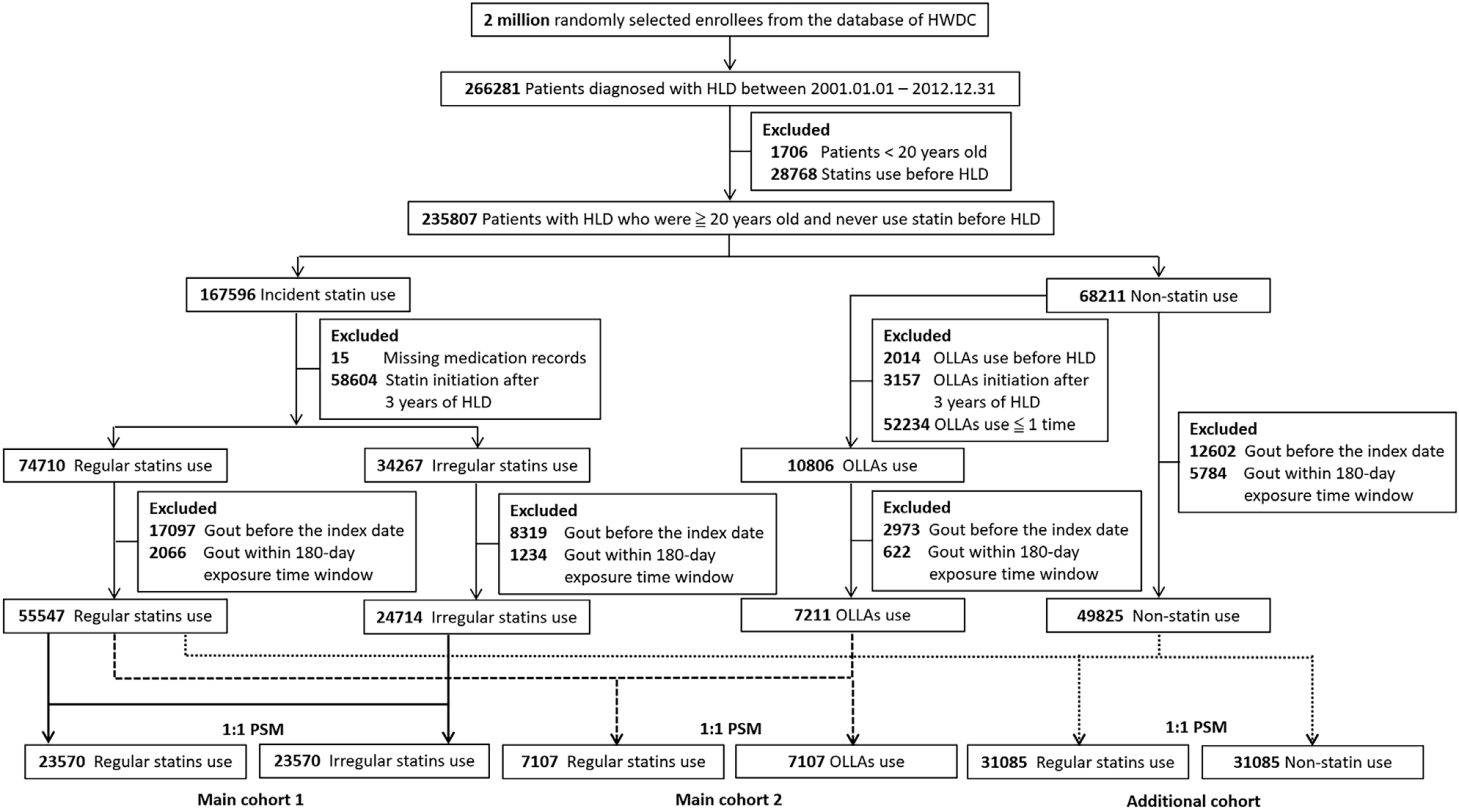
Flowchart of the enrollment of the study cohort. HWDC, Health and Welfare Data Science Center; HLD, hyperlipidemia; OLLA, other lipid-lowering agent; PSM, propensity score matching.

Figure 1 illustrates the selection process of the study population. Patients aged <20 years were excluded. To ensure the enrollment of new users, patients ever prescribed with statins before the hyperlipidemia diagnosis were excluded. In addition, patients diagnosed as having hyperlipidemia and initiated statins or OLLAs after 3 years of the diagnosis were excluded because the severity of hyperlipidemia might be dissimilar between later and earlier initiators. Moreover, patients with missing medication records were excluded. We divided statin initiators into regular statin users and irregular statin users in accordance with whether they met the criteria of regular use. To reduce confounding by indication and immortal time bias (Yoshida et al., 2015), irregular statin users and OLLA users were considered as active comparators. We then excluded patients with gout before the index date. To prevent the misclassification of outcomes, patients with gout in the 180-day exposure time window after the index date were excluded because earlier outcomes might be affected by certain circumstances before treatment (Lee, 2013). Propensity score matching (PSM) was applied to address the imbalance and create a new comparative cohort by excluding dissimilar samples (Franchetti, 2022). Each regular statin user was 1:1 randomly matched with an irregular statin user or an OLLA user on the basis of variables related to the likelihood of statin treatment, namely age, sex, calendar year of the index date (to adjust the effect of secular trend) (Seeger et al., 2005), comorbidities, medication use and lifestyle factors.

Although using non-user as a comparator might lead a bias of confounding by indication, the results might indicate treatment would be ineffective if a user group has a poor prognosis (Sharma et al., 2019). We still investigated the effect of statin use compared with that of no statin use. An additional cohort was included to compare the effect of regular statin use with that of no statin use. Non-statin users were patients never receiving any statins during the study period. The immortal time bias was considered in this additional study, and new index dates were assigned for non-statin users based on the prescription time-distribution of regular statin users (Zhou et al., 2005; Karim et al., 2016).

### 2.3 Definition of exposure

By using Anatomical Therapeutic Chemical (ATC) codes, patients who have ever received statins (C10AA) or OLLAs (C10AB, C10AC, C10AD, and C10AX) were identified from the database. Regular statin users were defined as those who received ≥2 prescriptions of statins in the outpatient clinic and consumed statins for ≥90 days within 1 year after the index date. Irregular statin users were defined as those not meeting the criteria of regular statin use. To identify the patients not occasionally using OLLAs, we excluded patients prescribed OLLA only once.

### 2.4 Covariates and confounders

Covariates for adjustment were related to gout, and they included demographic factors (age and sex), lifestyle factors, comorbidities, and medication use. Comorbidities included hypertension, DM, CHD, chronic heart failure, urolithiasis, chronic kidney disease, psoriasis, hypothyroidism, hyperthyroidism, anemia, menopause, and obstructive sleep apnea. Medications included thiazide diuretics, loop diuretics, aspirin, cytotoxic agents, pyrazinamide, ethambutol, ciclosporin, and tacrolimus. The use of medications exerting a pleotropic effect, namely metformin (Vazirpanah et al., 2019), or preventing gout, namely colchicine, urate-lowering therapy (ULT), and losartan (Wolff et al., 2015), were accounted for. Lifestyle factors, namely obesity, alcohol use, and tobacco use, were also considered. In clinical practice, some asymptomatic patients were prescribed ULT or colchicine for prevention or backup. Thus, the prescription of ULT or colchicine before the index date was used as a surrogate variable for the adjustment of the potential risk of gout. Diagnosis codes of comorbidities were identified if those were recorded ≥2 times during outpatient visits or once during hospitalization in the databases. However, lifestyle factors were rarely recorded as diagnosis in the NHI database. A record of these factors indicates their high severity. Thus, the diagnosis codes of lifestyle factors were identified if recorded once. Medication use was identified on the basis of a prescription of ≥28 days within 1 year before the index date.

### 2.5 Measurement of the primary outcome

The primary outcome was the incidence of gout. The diagnosis of gout obtained from outpatient and inpatient records was based on the *ICD-9-CM* code 274.X and *ICD-10-CM* code M10.X. To ensure the robustness of the outcome definition, we considered patients with related diagnosis codes of gout who were concomitantly prescribed colchicine or ULT within 30 days of the diagnosis date. In this study, death was defined as a censored event. The Cause of Death Database containing information regarding the cause and date of death was utilized to ensure deaths.

### 2.6 Measurement of the secondary outcome

The secondary outcome was dose- and duration-dependent associations between statin use and gout incidence. We used World Health Organization’s ATC code and the Defined Daily Dose (DDD) Index to explore the drug consumption during the follow-up period. The DDD of each regular statin user was based on the assumed average maintenance dose per day for modifying lipid levels in adults. In the dose-related analysis, the cumulative DDDs (cDDDs) were categorized into groups of <360, 360 to <720, 720 to <1080, and ≥1080. The duration of statin use was determined through the summation of all intervals during the follow-up period and divided into <3, 3 to <5, 5 to <7, and ≥7 years.

### 2.7 Statistical analysis

Baseline continuous variables are described as the mean ± standard deviation. Categorical variables are described as the percentage (%). A standardized mean difference (SMD) was calculated to determine the balance in covariates between regular statin users and two active comparators after PSM. An absolute value of SMD >0.10 indicated meaningful imbalance (Franchetti, 2022). The cumulative incidence curves of gout were plotted using the Kaplan-Meier method. The log-rank test was performed to examine differences between two curves. To determine the causal effect of statin use on the time-to-event outcomes of gout, a marginal Cox proportional hazard model was used to estimate hazard ratios (HRs) and 95% confidence intervals (CIs). In this model, a robust sandwich covariance matrix estimator was used to account for clustering and produce unbiased HRs, which have a precise standard error (Austin, 2014). In addition to crude estimation, the remaining imbalanced variables and all variables were adjusted in Cox regression models. Proportional hazards assumptions were tested, and no violation was observed. All data analyses were performed using SAS (version 9.4, SAS Institute Inc. Cary, NC, United States). Statistical significance was defined as a two-tailed *p*-value of <0.05.

## 3 Results

### 3.1 Baseline characteristics

Figure 1 presents the flowchart of the selection of the study population. We identified 55,547 patients with regular statin use, 24,714 patients with irregular statin use, and 7,211 patients with OLLA use. After PSM, 47,140 patients and 14,214 patients were recruited in main cohort 1 and main cohort 2, respectively. Table 1 lists the demographic characteristics of the participants. The regular statin users were older (mean age: 56.92 ± 11.94 years in 2 main cohorts). The proportion of male regular statin users was higher (25,458, 45.83%) in main cohort 1 but lower in main cohort 2. Apart from the period from the diagnosis to the index date in main cohort 2, all the covariates were well balanced after PSM. Overall, 4,375 (9.28%) patients developed gout [2,153 (9.13%) and 2,222 (9.43%) among regular and irregular statin users, respectively] in main cohort 1, and 1,374 (9.67%) patients developed gout [677 (9.53%) and 697 (9.81%) among regular statin users and OLLA users, respectively] in main cohort 2. The mean follow-up period between the regular (9.44 ± 3.82 years) and irregular (9.30 ± 3.92 years) statin users significantly differed (*p* < 0.05) in main cohort 1. A similar result (8.99 ± 3.85 years for regular statin users and 8.42 ± 3.98 years for OLLA users, *p* < 0.05) was observed in main cohort 2.

**TABLE 1:** Demographic characteristics of participants in 2 main cohorts.

Variables	Main cohort 1						Main cohort 2					
	Full cohort			1:1 PSM^a^ cohort			Full cohort			1:1 PSM^a^ cohort		
	Regular statin use	Irregular statin use	SMD^b^	Regular statin use	Irregular statin use n = 23570	SMD^b^	Regular statin use	OLLA use n = 7211	SMD^b^	Regular statin use	OLLA use n = 7107	SMD^b^
	n = 55547	n = 24714		n = 23570			n = 55547			n = 7107		
Age/year, mean ± SD	56.92 ± 11.94	55.60 ± 12.56	−0.11	55.90 ± 12.24	55.81 ± 12.32	−0.01	56.92 ± 11.94	52.34 ± 13.79	0.36	52.48 ± 13.56	52.41 ± 13.66	−0.01
Sex/male, n (%)	25458 (45.83)	10396 (42.07)	0.08	9914 (42.06)	9914 (42.06)	0.00	25458 (45.83)	4607 (63.89)	0.37	4525 (63.67)	4525 (63.67)	0.00
Period from diagnosis to the index date, mean days ± SD	195.2 ± 300.7	180.6 ± 293.8	−0.05	173.9 ± 287.7	183.4 ± 295.5	0.03	195.2 ± 300.7	118.1 ± 249.8	0.28	161.6 ± 276.5	118.7 ± 250.5	−0.16
**Comorbidities, n (%)**												
Hypertension	28837 (51.91)	9276 (37.53)	0.29	9161 (38.87)	9169 (38.90)	−0.00	28837 (51.91)	2773 (38.46)	−0.27	2698 (37.96)	2752 (38.72)	−0.02
Diabetes	19261 (34.68)	5741 (23.23)	0.25	5628 (23.88)	5644 (23.95)	−0.00	19261 (34.68)	1624 (22.52)	−0.27	1548 (21.78)	1605 (22.58)	−0.02
Coronary heart disease	9176 (16.52)	3171 (12.83)	0.10	3101 (13.16)	3102 (13.16)	−0.00	9176 (16.52)	676 (9.37)	−0.21	696 (9.79)	665 (9.36)	0.01
Chronic heart failure	1316 (2.37)	475 (1.92)	0.03	406 (1.72)	467 (1.98)	−0.02	1316 (2.37)	109 (1.51)	−0.06	94 (1.32)	107 (1.51)	−0.01
Urolithiasis	1178 (2.12)	491 (1.99)	0.01	370 (1.57)	433 (1.84)	−0.02	1178 (2.12)	167 (2.32)	0.01	108 (1.52)	165 (2.32)	−0.05
Chronic kidney disease	878 (1.58)	351 (1.42)	0.01	306 (1.30)	342 (1.45)	−0.01	878 (1.58)	116 (1.61)	0.00	88 (1.24)	111 (1.56)	−0.03
Psoriasis	161 (0.29)	64 (0.26)	0.01	51 (0.22)	62 (0.26)	−0.01	161 (0.29)	22 (0.31)	0.00	17 (0.24)	22 (0.31)	−0.01
Hypothyroidism	403 (0.73)	165 (0.67)	0.01	123 (0.52)	161 (0.68)	−0.02	403 (0.73)	27 (0.37)	−0.05	28 (0.39)	26 (0.37)	0.00
Hyperthyroidism	424 (0.76)	152 (0.62)	0.02	117 (0.50)	149 (0.63)	−0.02	424 (0.76)	27 (0.37)	−0.05	21 (0.30)	26 (0.37)	−0.01
Anemia	1125 (2.03)	549 (2.22)	−0.01	424 (1.80)	514 (2.18)	−0.03	1125 (2.03)	146 (2.02)	−0.00	95 (1.34)	132 (1.86)	−0.04
Menopause	1789 (3.22)	1074 (4.35)	−0.06	959 (4.07)	1039 (4.41)	−0.02	1789 (3.22)	144 (2.00)	−0.08	122 (1.72)	144 (2.03)	−0.02
Obstructive sleep apnea	48 (0.09)	23 (0.09)	−0.00	13 (0.06)	20 (0.08)	−0.01	48 (0.09)	6 (0.08)	−0.00	4 (0.06)	6 (0.08)	−0.01
**Medications, n (%)**												
Thiazide diuretics	5582 (10.05)	2672 (5.36)	0.11	1731 (7.34)	1722 (7.31)	0.00	5582 (10.05)	537 (7.45)	−0.09	471 (6.63)	527 (7.42)	−0.03
Loop diuretics	1814 (3.27)	1040 (2.09)	0.03	599 (2.54)	637 (2.70)	−0.01	1814 (3.27)	198 (2.75)	−0.03	158 (2.22)	189 (2.66)	−0.03
Aspirin	12355 (22.24)	4288 (8.61)	0.16	3982 (16.89)	3839 (16.29)	0.02	12355 (22.24)	892 (12.37)	−0.26	929 (13.07)	882 (12.41)	0.02
Cytotoxic agents	216 (0.39)	232 (0.47)	0.01	62 (0.26)	72 (0.31)	−0.01	216 (0.39)	24 (0.33)	−0.01	20 (0.28)	24 (0.34)	−0.01
Pyrazinamide	18 (0.03)	27 (0.05)	0.00	7 (0.03)	8 (0.03)	−0.00	18 (0.03)	−	−0.00	3 (0.04)	−	0.01
Ethambutol	76 (0.14)	77 (0.15)	0.00	36 (0.15)	32 (0.14)	0.00	76 (0.14)	10 (0.14)	0.00	9 (0.13)	10 (0.14)	−0.00
Ciclosporin	30 (0.05)	25 (0.05)	0.01	13 (0.06)	7 (0.03)	0.01	30 (0.05)	3 (0.04)	−0.01	3 (0.04)	−	0.01
Tacrolimus	12 (0.02)	6 (0.01)	0.00	3 (0.01)	4 (0.02)	−0.00	12 (0.02)	−	0.00	−	−	−0.01
Metformin	13536 (24.37)	4005 (8.04)	0.25	3512 (14.90)	3494 (14.82)	0.00	13536 (24.37)	1062 (14.73)	−0.25	1018 (14.32)	1046 (14.72)	−0.01
Colchicine	166 (0.30)	90 (0.18)	0.02	64 (0.27)	51 (0.22)	0.01	166 (0.30)	22 (0.31)	0.00	11 (0.15)	21 (0.30)	−0.03
Urate-lowering agents	764 (1.38)	322 (0.65)	0.01	273 (1.16)	309 (1.31)	−0.01	764 (1.38)	124 (1.72)	0.03	98 (1.38)	115 (1.62)	−0.02
Losartan	3756 (6.76)	1440 (2.89)	0.14	906 (3.84)	898 (3.81)	0.00	3756 (6.76)	297 (4.12)	−0.12	287 (4.04)	291 (4.09)	−0.00
**Lifestyle factors, n (%)**												
Obesity	647 (1.16)	295 (1.19)	−0.00	229 (0.97)	291 (1.23)	−0.00	647 (1.16)	77 (1.07)	−0.01	60 (0.84)	76 (1.07)	−0.02
Alcohol use	49 (0.09)	28 (0.11)	−0.01	17 (0.07)	20 (0.08)	−0.00	49 (0.09)	22 (0.31)	0.05	9 (0.13)	11 (0.15)	−0.01
Tobacco use	339 (0.61)	153 (0.62)	−0.00	106 (0.45)	149 (0.63)	−0.02	339 (0.61)	57 (0.79)	0.02	30 (0.42)	54 (0.76)	−0.04

SD, standard deviation; n, number; OLLA, other lipid-lowering agent.

^a^ PSM, propensity score matching, matched by the age range, sex, calendar year of the index date, 12 comorbidities, 12 medication use, and 3 lifestyle factors.

^b^ SMD, standardized mean difference, the difference in means or proportions divided by the standard error; imbalance defined as an absolute value of >0.10.

−According to the regulation of HWDC, data less than 3 units could not be disclosed.

### 3.2 Primary outcome

**FIGURE 2:**
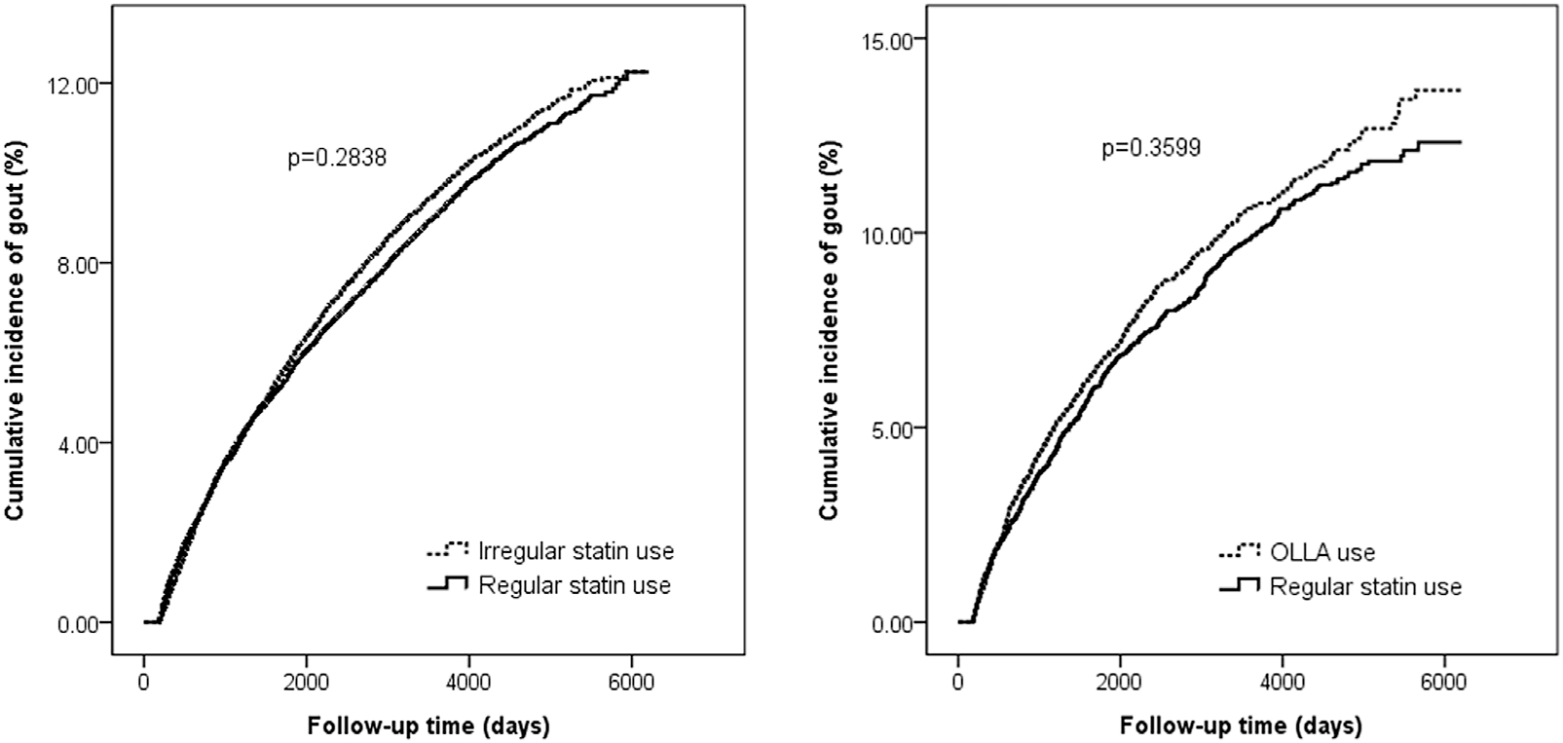
Cumulative incidence of gout in 2 main cohorts. OLLA, other lipid-lowering agent.

Figure 2 illustrates the cumulative incidence of gout. No significant difference in the Kaplan-Meier curves was noted between the regular statin users and irregular statin users (*p* = 0.28) or OLLA users (*p* = 0.36). In main cohort 1, the incidence rate of gout was 9.68 per 1,000 person-years (95% CI, 9.28–10.10) and 10.14 per 1,000 person-years (95% CI, 9.72–10.56) among the regular and irregular statin users, respectively. In main cohort 2, the incidence rate of gout was 10.59 per 1,000 person-years (95% CI, 9.81–11.41) and 11.64 per 1,000 person-years (95% CI, 10.80–12.53) among the regular statin users and OLLA users, respectively.

In the risk analysis, a non-significant reduction was observed in both the study cohorts (crude HR, 0.96; 95% CI, 0.90–1.02 in main cohort 1 and crude HR, 0.92; 95% CI, 0.83–1.02 in main cohort 2; Table 2). Even the remaining imbalanced or all covariates were adjusted for in different regression models, and the effect size of the adjusted HR (aHR) was similar to that of crude HR (Table 2). In the sensitivity analysis, we considered a follow-up period of 5 years for each individual instead of follow-up until the end of the study, and univariable analysis results still revealed non-significant effects (crude HR, 0.96; 95%, CI, 0.89–1.03 in main cohort 1 and crude HR, 0.94; 95% CI, 0.82–1.07 in main cohort 2; Table 2). The multivariable analysis produced similar results (Table 2).

**TABLE 2:** Risk and sensitivity analyses for regular statin use and the gout risk in 2 main cohorts.

Model	Main cohort 1			Main cohort 2		
	Regular statin use	Irregular statin use	*p*-value	Regular statin use	OLLA use	*p*-value
**Follow-up until the end of study**						
Unadjusted, crude HR (95% CI)	0.96 (0.90–1.02)	1	0.14	0.92 (0.83–1.02)	1	0.12
Model 1, adjusted HR (95% CI)^a^	NA	NA	NA	0.93 (0.84–1.03)	1	0.16
Model 2, adjusted HR (95% CI)^b^	0.95 (0.90–1.01)	1	0.10	0.94 (0.84–1.04)	1	0.22
**Follow-up for 5 years**						
Unadjusted, crude HR (95% CI)	0.96 (0.89–1.03)	1	0.27	0.94 (0.82–1.07)	1	0.32
Model 1, adjusted HR (95% CI)^a^	NA	NA	NA	0.94 (0.83–1.07)	1	0.35
Model 2, adjusted HR (95% CI)^b^	0.96 (0.88–1.03)	1	0.23	0.95 (0.84–1.09)	1	0.47

HR, hazard ratio; CI, confidence interval; NA, not available.

^a^ Model 1: adjusted for covariates that remained imbalanced after PSM.

For main cohort 1: NA, all covariates were balanced.

For main cohort 2: adjusted for covariate “period from the diagnosis to the index date”.

^b^ Model 2: adjusted for all covariates.

### 3.3 Secondary outcome

We examined the dose-dependent association between regular statin use and gout risk (Table 3). In the dose-related analysis, the <360 cDDD group exhibited a harmful effect in both main cohort 1 and 2 (crude HR, 2.00; 95% CI, 1.83–2.18 and crude HR, 1.91; 95% CI, 1.64–2.22, respectively). However, when the cumulative exposure increased to 360 to <720 cDDDs, the effect size was observed to be neutral. A protective effect was observed when the patients used ≥720 cDDDs in both the cohorts (crude HR, 0.60; 95% CI, 0.50–0.72 in main cohort 1 and crude HR, 0.47; 95% CI, 0.34–0.65 in main cohort 2). Consistent trends were noted in different adjustment models (Table 3). In the duration-related analysis, short-term use (<3 years) demonstrated a harmful effect in both main cohort 1 and 2 (crude HR, 1.72; 95% CI, 1.59–1.86 and crude HR, 1.73; 95% CI, 1.52–1.97, respectively). However, a significant protective effect was noted when the patients received statins for ≥3 years in both the cohorts (crude HR, 0.76; 95% CI, 0.64–0.90 in main cohort 1 and crude HR, 0.49; 95% CI, 0.36–0.66 in main cohort 2). Similar results were observed in different adjustment models (Table 3). After changing the follow-up period to 5 years for each individual in the sensitivity analysis, we still observed dose- and duration-dependent associations, even in the adjustment models (Table 3).

**TABLE 3:** Dose- and duration-related analyses and sensitivity analyses of statin use and the gout risk in 2 main cohorts.

		Main cohort 1					Main cohort 2			
	Regular statin use	Irregular statin use	Crude HR (95%CI)	aHR^a^ (95%CI)	aHR^b^ (95%CI)	Regular statin use	OLLA use	Crude HR (95%CI)	aHR^a^ (95%CI)	aHR^b^ (95%CI)
	Event n/total n (%)					Event n/total n (%)				
**Follow-up until the end of study**										
<360 cDDDs	1350/7728 (17.47)	754/7728 (9.76)	2.00 (1.83–2.18)	NA	2.03 (1.86–2.22)	446/2488 (17.93)	250/2488 (10.05)	1.91 (1.64–2.22)	1.91 (1.64–2.22)	1.96 (1.68–2.29)
360 to <720 cDDDs	363/3953 (9.18)	331/3953 (8.37)	1.08 (0.93–1.25)	NA	1.08 (0.93–1.26)	121/1252 (9.66)	116/1252 (9.27)	0.99 (0.77–1.27)	1.00 (0.78–1.29)	1.00 (0.77–1.30)
720 to <1080 cDDDs	175/3102 (5.64)	273/3102 (8.80)	0.60 (0.50–0.72)	NA	0.57 (0.47–0.69)	53/941 (5.63)	100/941 (10.63)	0.47 (0.34–0.65)	0.48 (0.34–0.66)	0.48 (0.34–0.67)
≥1080 cDDDs	265/8787 (3.02)	864/8787 (9.83)	0.27 (0.24–0.31)	NA	0.27 (0.23–0.30)	57/2426 (2.35)	231/2426 (9.52)	0.21 (0.16–0.27)	0.21 (0.16–0.28)	0.21 (0.15–0.27)
**Follow-up for 5 years**										
<360 cDDDs	1046/12593 (8.31)	770/12593 (6.11)	1.38 (1.26–1.52)	NA	1.38 (1.26–1.52)	350/3829 (9.14)	265/3829 (6.92)	1.32 (1.13–1.55)	1.32 (1.13–1.55)	1.35 (1.15–1.59)
360 to <720 cDDDs	194/5930 (3.27)	319/5930 (5.38)	0.58 (0.49–0.70)	NA	0.59 (0.49–0.70)	76/1795 (4.23)	107/1795 (5.96)	0.66 (0.50–0.89)	0.66 (0.49–0.89)	0.66 (0.49–0.89)
720 to <1080 cDDDs	44/2895 (1.52)	161/2895 (5.56)	0.26 (0.19–0.36)	NA	0.23 (0.17–0.33)	18/831 (2.17)	55/831 (6.62)	0.30 (0.18–0.50)	0.31 (0.18–0.51)	0.32 (0.19–0.55)
≥1080 cDDDs	23/2152 (1.07)	100/2152 (4.65)	0.22 (0.14–0.34)	NA	0.21 (0.14–0.33)	4/652 (0.61)	38/652 (5.83)	0.10 (0.04–0.28)	0.10 (0.04–0.28)	0.09 (0.03–0.23)
**Follow-up until the end of study**										
<3 years	1660/10965 (15.14)	1041/10965 (9.49)	1.72 (1.59–1.86)	NA	1.73 (1.60–1.87)	567/3554 (15.95)	341/3554 (9.59)	1.73 (1.52–1.97)	1.74 (1.52–1.98)	1.78 (1.56–2.03)
3 to <5 years	249/3772 (6.60)	310/3772 (8.22)	0.76 (0.64–0.90)	NA	0.76 (0.64–0.90)	61/1178 (5.18)	112/1178 (9.51)	0.49 (0.36–0.66)	0.50 (0.37–0.68)	0.50 (0.37–0.68)
5 to <7 years	126/3282 (3.84)	270/3282 (8.23)	0.43 (0.35–0.52)	NA	0.41 (0.33–0.51)	24/983 (2.44)	95/983 (9.66)	0.22 (0.14–0.34)	0.22 (0.14–0.34)	0.21 (0.14–0.33)
≥7 years	118/5551 (2.13)	601/5551 (10.83)	0.17 (0.14–0.21)	NA	0.17 (0.14–0.20)	25/1392 (1.80)	149/1392 (10.70)	0.14 (0.09–0.21)	0.14 (0.09–0.21)	0.13 (0.09–0.20)
**Follow-up for 5 years**										
<1 year	817/8924 (9.16)	548/8924 (6.14)	1.54 (1.38–1.71)	NA	1.54 (1.38–1.72)	279/2775 (10.05)	182/2775 (6.56)	1.56 (1.30–1.88)	1.55 (1.29–1.87)	1.60 (1.33–1.93)
1 to <3 years	407/8235 (4.94)	475/8235 (5.77)	0.83 (0.73–0.95)	NA	0.83 (0.72–0.95)	143/2478 (5.77)	163/2478 (6.58)	0.83 (0.67–1.04)	0.84 (0.67–1.05)	0.84 (0.67–1.06)
≥3 years	83/6411 (1.29)	327/6411 (5.10)	0.24 (0.19–0.30)	NA	0.24 (0.19–0.30)	26/1854 (1.40)	120/1854 (6.47)	0.20 (0.13–0.30)	0.20 (0.13–0.31)	0.20 (0.13–0.31)

cDDD, cumulative defined daily dose; HR, hazard ratio; CI, confidence interval; n, number; NA, not available.

^a^ Adjusted for covariates that remained imbalanced after PSM.

^b^ Adjusted for all covariates.

For main cohort 1: NA, all covariates were balanced.

For main cohort 2: adjusted for covariate “period from the diagnosis to the index date”.

^c^ Comparison group for main cohort 1 is irregular statin use, and main group 2 is other lipid-lowering agent (OLLA) use.

### 3.4 Additional analysis

To interpret the relative benefit of statin use compared with that of no statin use, we performed an additional analysis including 62,170 patients (Figure 1). As presented in Supplementary Table S1 in the online repository, the proportions of older patients, female patients, those with chronic diseases (including hypertension, DM, and CHD), and medication use (including thiazide diuretics, aspirin, metformin, and losartan) were higher in the regular statin users, and three covariates (hypertension, DM, and metformin use) were still imbalanced after PSM. In the primary analysis, crude and adjusted estimates indicated gout risk was 1.2-fold higher among the regular statin users than in the statin non-users in both the full-length follow-up and 5-year follow-up (Supplementary Table S2). However, a significant protective effect was observed in the dose- or duration-dependent analysis. The pattern was also observed in the 5-year sensitivity analysis (Supplementary Table S3).

## 4 Discussion

To the best of our knowledge, this is the first real-world study to examine the association between regular statin use and gout risk in patients with hyperlipidemia. In this nationwide retrospective cohort study, regular statin use non-significantly reduced the incidence of gout compared with irregular statin use or OLLA use. However, the protective effect appeared with increases in the cumulative exposure dose and treatment duration. The dose- and duration-dependent patterns were observed not only during the full-length follow-up but during the 5-year follow-up.

In the immunopathogenesis of gout, the needle-shaped MSU can act as a danger signal and trigger an innate immune response (Franklin et al., 2016; Gong et al., 2020). Moreover, MSU triggers the cellular inflammatory response though two-signal pathways. Signal 1 activates the Toll-like receptor (TLR) and its intracellular adapter protein MyD88 and subsequently enhances the expression of NF-κB and the production of the functional components of the NLRP3 inflammasome. Signal 2 activates the phagocytosis of MSU crystals in macrophages. Then, lysosomal rupture causes damage to organelles and mitochondrial reactive oxygen species (ROS) release. ROS triggers the assembly of the NLRP3 inflammasome, and then promotes caspase-1 synthesis and inflammatory interleukin (IL)-1β formation (So and Martinon, 2017). Research on cardiovascular or non-cardiovascular complications has indicated statins can inhibit inflammasome formation by suppressing the TLR4/MyD88/NF-κB pathway and attenuate IL-1β production in animal or cellular models. Although gout was not examined in these experimental models, other inflammatory diseases have been investigated (Bahrami et al., 2018). A recent study indicated that similar to MSU, cholesterol crystals are involved in the two-signal inflammation mechanism, and statins can suppress TLR4/MyD88/NF-κB signaling (Koushki et al., 2020). In the current study, the decrease in the incidence of gout resulting from long-term statin use might also involve the inhibition of TLR4/MyD88/NF-κB signaling.

Observational studies have indicated statin use can reduce the risks of some inflammatory and immune diseases or improve the survival of patients with these diseases including inflammatory bowel disease (Crockett et al., 2012; Ungaro et al., 2016), chronic obstructive pulmonary disease (Raymakers et al., 2017; Lei et al., 2020), systemic lupus erythematosus (Yu et al., 2015; De Jong et al., 2017), rheumatoid arthritis (Schoenfeld et al., 2016; Tascilar et al., 2016), psoriasis (Wolkenstein et al., 2009; Chodick et al., 2015), and ankylosing spondylitis (Oza et al., 2017). Our study revealed the anti-inflammatory property of statins in gout, especially for a higher dose or long-term exposure of statins. To date, only two studies examined the association of statin use with gout. One population-based cohort study using The Health Improvement Network database of UK general practices revealed statin use resulted in a 16% reduction in mortality in the gout population (HR, 0.84; 95% CI, 0.79–0.89) (Keller et al., 2018). Although the other population-based cohort study investigating the effectiveness of statins for primary prevention in gout population did not reveal a significant decrease in the incidence of CHD (HR, 0.84; 95% CI, 0.60–1.19), ischemic stroke (HR, 0.68; 95% CI, 0.44–1.05) and all-cause mortality (HR, 0.87; 95% CI, 0.67–1.12), the precision of effect size might be affected by the small sample size in this study (Garcia-Gil et al., 2019).

Clinical or *in vitro* studies (Crockett et al., 2012; Gonçalves et al., 2019; Chang et al., 2021; Prado et al., 2021) have indicated the anti-inflammatory property of statins is dose- or duration-dependent; this finding is consistent with that of our secondary analyses. Even in our primary analysis, regular statin use non-significantly reduced gout risk compared with irregular statin use or OLLA use. A potential explanation for this finding is that the group of regular statin users might include too many patients receiving lower doses (>32% of patients received <360 cDDD, Table 3) or receiving statins for a short duration (>45% of patients received statins for <3 years, Table 3).

Apart from the anti-inflammatory property, two types of commonly prescribed statins, atorvastatin and simvastatin but not other statins, have been reported to reduce serum uric acid (Derosa et al., 2016). Therefore, statins might reduce the gout incidence *via* this mechanism. However, a puzzle was observed that not all patients with hyperuricemia suffer from gout (Bardin and Richette, 2014). Some patients with gout can have normuricemia (Schlesinger et al., 2009; Lee et al., 2020), and reducing serum uric acid may not be the only way to eliminate gout flares (Zhang, 2021). Whether the effectiveness of statin on gout incidence is attributed to lowering the serum uric acid needs to be proved.

In a longitudinal cohort study, the follow-up duration of the intervention group was generally longer than that of the comparator group, and this attrition bias led to a biased outcome estimation (Sharma et al., 2019). To diminish this bias, we equally reduced the follow-up period to 5 years for each individual. We observed consistent results in both the primary risk estimation and secondary dose- and duration-dependent analyses. The findings indicated that a long follow-up period did not bias our major findings.

To improve the quality of comparison, choosing an active comparator who received a drug with the same or similar indication results in the similarity of two groups. However, if the drug of interest is the most commonly used in a given condition, its alternatives (similar to statin comparators) are infrequently used. Selecting an active comparator becomes challenging. Furthermore, it is difficult to interpret the relative benefit of the drug of interest and an active comparator, where the effect of the active comparator is still unknown. Having multiple comparators, including an active comparator and a non-user group, may be a favorable option for interpreting findings (Yoshida et al., 2015). Thus, we selected two groups of active comparators with a likely disease status and conducted an additional analysis for the comparison of statin use and nonuse.

The definition of outcomes in our study was carefully considered. Four criteria were applied for determining gout in an epidemiological study conducted in Taiwan (Chen et al., 2012), and this definition might lead to the loss of real patients with gout for whom ULT was not prescribed by rheumatologists. Considering only one diagnosis code of gout or ULT initiation as a definition might not be adequately accurate. We considered that long-term ULT should be initiated after the diagnosis, and we used one diagnosis code plus an antigout (colchicine or ULT) prescription within 30 days of the diagnosis as an outcome, which might be more accurate and feasible for the definition of gout.

Because of the lack of random allocation for the feature of an observational study, we used the PSM to recreate a well-balanced cohort. If measured and unmeasured covariates are all accounted for, the treatment effect can be estimated by comparing groups as if the experiment were a randomized trial (Franchetti, 2022). In this study, the incident cases of hyperlipidemia and new user design were applied to prevent the prevalent user bias and reduce reversed causation. The initiation of the follow-up was aligned from the index date in all the groups. To prevent outcome misclassification, we set a 180-day exposure time window after the index date and excluded the outcome determining during this period because earlier outcomes might be affected by certain circumstances before the treatment.

The LGTD 2000 used as a data source in this study consisted of updated records regarding prescriptions and diagnoses. Thus, we adjusted the medication use, which was ignored in previous studies. Furthermore, we considered medications exerting pleiotropic effect (such as metformin) or exhibiting a property of hypouricemia (such as losartan) and adjusted these covariates in regression models.

This study has some limitations. Lack of laboratory data, such as that on the low-density lipoprotein cholesterol level, is a limitation of utilizing the NHI database. However, we calculated the medication initiation period from the diagnosis to the index date to represent the severity of hyperlipidemia. Theoretically, colchicine and ULT cannot be used before gout diagnosis (Yu et al., 2018). Thus, if these medicines are prescribed for backup, these patients might be more likely to have gout. We adjusted these two medications as the surrogate covariates of the potential risk of gout. Lifestyle factors (e.g., consumption of meat or seafood) were associated with the risk of gout and were not recorded in this claims database. However, we adjusted other lifestyle factors (e.g., obesity, alcohol, and tobacco use), and those were associated with gout. Other factors, such as the family history or genotype, were not recorded in this database. For patients with real outcomes but without seeking medical care, personal problems, such as these behaviors or medication adherence, were not recorded. In addition, we did not analyze the outcome from stratifying the statins into different types, and the overall outcome might be attributed to certain types, different potency or lipophilicity of statins. Finally, the generalizability of study findings is limited because we excluded a part of unmatched patients. Although these patients might be systematically different from matched patients, they might limit the representativeness of the whole population (Allan et al., 2020).

In conclusion, this nationwide, population-based cohort study revealed that regular statin use non-significantly reduced the risk of gout in the hyperlipidemia population. However, a protective benefit of statins was observed for a higher dose and long-term therapy. Clinically, patients with hyperlipidemia who are prescribed statins should be highly encouraged for continual and adherent use, and this might be advantageous for gout prevention. Future studies for elucidating the molecular mechanism through which statins inhibit gout flare in cellular levels or animal models, verifying the effectiveness of statins in reducing the risk of gout between diverse races or populations, or even confirming the study results by utilizing other administrative databases or electronic medical records are recommended.

## Data Availability

The datasets presented in this article are not readily available because the regulation of Health and Welfare Data Science Center (HWDC), datasets or raw data generated or analyzed from National Health Insurance Research Database (NHIRD) could not be shared in public in order to protect patients’ confidentiality, which is used under license. If request of details is needed, formal requisition can be sent to the HWDC. Requests to access the datasets should be directed to https://dep.mohw.gov.tw/DOS/cp-2516-59203-113.html.
