# Supplementary material for: Association between statin use and the risk of gout in patients with hyperlipidemia: A population-based cohort study
Source: Front Pharmacol. 2023 Feb 15;14:1096999. doi: 10.3389/fphar.2023.1096999 (PMC9975165; doi:10.3389/fphar.2023.1096999)
Supplement: Supplementary file 1 [file DataSheet1.PDF]

Supplementary Table S1. Demographic characteristics of participants in the additional cohort

Variables	Full cohort			1:1 PSM[b] cohort		
	Regular statin use n = 55547	No statin use n = 49825	SMD[a]	Regular statin use n = 31085	No statin use n = 31085	SMD[a]
Age/year, mean ± SD	56.92 ± 11.94	51.84 ± 14.70	0.38	56.56±12.80	56.08±12.84	0.04
Sex/male, n (%)	25458 (45.83)	24946 (50.07)	0.08	14270 (47.35)	14270 (47.35)	0.00
**Comorbidities, n (%)**						
Hypertension	28837 (51.91)	15020 (30.15)	−0.45	10731 (34.52)	13603 (43.76)	0.19
Diabetes	19261 (34.68)	7245 (14.54)	−0.48	8275 (26.62)	6489 (20.88)	-0.14
Coronary heart disease	9176 (16.52)	4027 (8.08)	−0.26	3855 (12.40)	3544 (11.40)	-0.03
Chronic heart failure	1316 (2.37)	855 (1.72)	−0.05	858 (2.76)	612 (1.97)	-0.05
Urolithiasis	1178 (2.12)	1043 (2.09)	−0.00	532 (1.71)	619 (1.99)	0.02
Chronic kidney disease	878 (1.58)	562 (1.13)	−0.04	565 (1.82)	415 (1.34)	-0.04
Psoriasis	161 (0.29)	137 (0.27)	−0.00	74 (0.24)	81 (0.26)	0.00
Hypothyroidism	403 (0.73)	365 (0.73)	0.00	314 (1.01)	270 (0.87)	-0.01
Hyperthyroidism	424 (0.76)	502 (1.01)	0.03	249 (0.80)	298 (0.96)	0.02
Anemia	1125 (2.03)	1314 (2.64)	0.04	715 (2.30)	782 (2.52)	0.01
Menopause	1789 (3.22)	1699 (3.41)	0.01	1012 (3.26)	1144 (3.68)	0.02
Obstructive sleep apnea	48 (0.09)	38 (0.08)	−0.00	17 (0.05)	30 (0.10)	0.02
**Medications, n (%)**						
Thiazide diuretics	5582 (10.05)	2672 (5.36)	−0.18	3910 (10.26)	2344 (7.54)	-0.10
Loop diuretics	1814 (3.27)	1040 (2.09)	−0.07	1222 (3.93)	755 (2.43)	-0.09
Aspirin	12355 (22.24)	4288 (8.61)	−0.38	4926 (15.85)	3917 (12.60)	-0.09
Cytotoxic agents	216 (0.39)	232 (0.47)	0.01	135 (0.43)	153 (0.49)	0.01
Pyrazinamide	18 (0.03)	27 (0.05)	0.01	8 (0.03)	15 (0.05)	0.01
Ethambutol	76 (0.14)	77 (0.15)	0.00	59 (0.19)	50 (0.16)	-0.01
Ciclosporin	30 (0.05)	25 (0.05)	−0.00	18 (0.06)	17 (0.05)	-0.00
Tacrolimus	12 (0.02)	6 (0.01)	−0.01	6 (0.02)	6 (0.02)	0.00
Metformin	13536 (24.37)	4005 (8.04)	−0.45	5021 (16.15)	3667 (11.80)	-0.13
Colchicine	166 (0.30)	90 (0.18)	−0.02	99 (0.32)	66 (0.21)	-0.02
Urate-lowering agents	764 (1.38)	322 (0.65)	−0.07	448 (1.44)	258 (0.83)	-0.06
Losartan	3756 (6.76)	1440 (2.89)	−0.18	1181 (3.80)	1309 (4.21)	0.02
**Lifestyle factors, n (%)**						
Obesity	647 (1.16)	778 (1.56)	0.03	401 (1.29)	365 (1.17)	-0.01
Alcohol use	49 (0.09)	94 (0.19)	0.03	36 (0.12)	43 (0.14)	0.01
Tobacco use	339 (0.61)	234 (0.47)	−0.02	184 (0.59)	203 (0.65)	0.01

NA, not available; SD, standard deviation; n, number.
[a]SMD, standardized mean difference, the difference in means or proportions divided by the standard error; imbalance defined as an absolute value of >0.10.
[b]PSM, propensity score matching, matched by the age range, sex, calendar year of the index date, 12 comorbidities, 12 medication use and 3 lifestyle factors.

Supplementary Table S2. Risk and sensitivity analyses for regular statin use and the gout risk in the additional cohort

	Model	Additional cohort		
		Regular statin use	No statin use	p value
Follow-up until the end of study	Unadjusted, crude HR (95% CI)	1.24 (1.17-1.31)	1	<0.01*
	Model 1, adjusted HR (95% CI)[a]	1.30 (1.23-1.38)	1	<0.01*
	Model 2, adjusted HR (95% CI)[b]	1.22 (1.15-1.29)	1	<0.01*
Follow-up for 5 years	Unadjusted, crude HR (95% CI)	1.21 (1.13-1.30)	1	<0.01*
	Model 1, adjusted HR (95% CI)[a]	1.28 (1.19-1.38)	1	<0.01*
	Model 2, adjusted HR (95% CI)[b]	1.20 (1.11-1.29)	1	<0.01*

HR, hazard ratio; CI, confidence interval; NA, not available, *$p < 0.05$.
[a]Model 1: adjusted for covariates that remained imbalanced (hypertension, diabetes, and metformin use) after PSM.
[b]Model 2: adjusted for all covariates.

Supplementary Table S3. Dose- and duration-related analyses and sensitivity analyses of statin use and gout risk in the additional cohort

.	Regular statin use	No statin use	Crude HR (95%CI)	aHR[a] (95%CI)	aHR[b] (95%CI)
	Event n/total n (%)				
**Follow-up until the end of study**					
<360 cDDDs	1752/10656 (16.44)	720/10656 (6.76)	2.60 (2.38-2.83)	2.71 (2.49-2.96)	2.62 (2.40-2.85)
360 to <720 cDDDs	496/5282 (9.39)	322/5282 (6.10)	1.45 (1.26-1.67)	1.54 (1.33-1.77)	1.43 (1.24-1.65)
720 to <1080 cDDDs	228/4163 (5.48)	271/4163 (6.51)	0.73 (0.62-0.87)	0.79 (0.66-0.94)	0.72 (0.60-0.87)
≥1080 cDDDs	310/10984 (2.82)	815/10984 (7.42)	0.32 (0.28-0.36)	0.34 (0.30-0.38)	0.30 (0.27-0.35)
**Follow-up for 5 years**					
<360 cDDDs	1353/16336 (8.28)	734/16336 (4.49)	1.87 (1.71-2.04)	1.96 (1.79-2.14)	1.85 (1.69-2.03)
360 to <720 cDDDs	268/7847 (3.42)	349/7847 (4.45)	0.73 (0.62-0.85)	0.78 (0.66-0.91)	0.73 (0.62-0.86)
720 to <1080 cDDDs	69/3965 (1.74)	192/3965 (4.84)	0.34 (0.26-0.44)	0.36 (0.27-0.47)	0.33 (0.25-0.43)
≥1080 cDDDs	32/2937 (1.09)	118/2937 (4.02)	0.26 (0.17-0.38)	0.27 (0.18-0.40)	0.25 (0.17-0.37)
**Follow-up until the end of study**					
<3 years	2195/14972 (14.66)	1001/14972 (6.69)	2.27 (2.11-2.45)	2.39 (2.21-2.57)	2.27 (2.10-2.45)
3 to <5 years	309/4986 (6.20)	311/4986 (6.24)	0.88 (0.75-1.02)	0.92 (0.78-1.08)	0.84 (0.72-0.99)
5 to <7 years	152/4520 (3.36)	276/4520 (6.11)	0.47 (0.39-0.57)	0.50 (0.41-0.61)	0.45 (0.37-0.55)
≥7 years	130/6607(1.97)	540/6607 (8.17)	0.20 (0.16-0.24)	0.21 (0.17-0.25)	0.19 (0.16-0.23)
**Follow-up for 5 years**					
<1 year	1050/11621 (9.04)	524/11621 (4.51)	2.05 (1.85-2.28)	2.17 (1.95-2.40)	2.04 (1.84-2.27)
1 to <3 years	560/10730 (5.22)	483/10730 (4.50)	1.12 (0.99-1.27)	1.18 (1.04-1.33)	1.10 (0.97-1.24)
≥3 years	112/8734 (1.28)	386/8734 (4.42)	0.27 (0.22-0.33)	0.29 (0.23-0.35)	0.27 (0.22-0.33)

cDDD, cumulative defined daily dose; HR, hazard ratio; CI, confidence interval; n, number; NA, not available.

[a]Adjusted for covariates that remain imbalanced (hypertension, diabetes, and metformin use) after PSM.

[b]Adjusted for all covariates.
